# A core-scale reconstructing method for shale

**DOI:** 10.1038/s41598-019-39442-5

**Published:** 2019-03-13

**Authors:** Lili Ji, Mian Lin, Gaohui Cao, Wenbin Jiang

**Affiliations:** 1 Key Laboratory for Mechanics in Fluid Solid Coupling Systems, Institute of Mechanics, Chinese Academy of Sciences, Beijing, 100190 China; 2 University of Chinese Academy of Sciences, Beijing, 100190 China

## Abstract

Characterization of shale cores with low and anisotropic permeability is complicated, due to the presence of multiscale pore structure and thin layers, and defies conventional methods. To accurately reproduce the morphology of multiscale pore structure of the shale core, a novel core-scale reconstructing method is proposed to reconstruct 3D digital-experimental models by means of the combination of SEM, EDS images, nitrogen adsorption and pressure pulse decay experiment result. In this method, the multiscale and multicomponent reconstructing algorithm is introduced to build the representative multiscale model for each layer, which can describe the complex 3D structures of nano organic pores, micro-nano inorganic pores, micro slits and several typical minerals. Especially, to reproduce the realistic morphology for shale, the optimization algorithm based on simulated annealing algorithm uses the experimental data as constrain conditions to adjust and optimize the model for each layer. To describe the bedding characteristics of the shale core, bedding fractures are constructed by analysis of the mineral distribution in the interface of two layers, and then the representative models for different layers are integrated together to obtain the final core-scale digital-experimental model. Finally, the model is validated by computing its morphological and flow properties and comparing them with those of the actual 3D shale sample. This method provide a way for systematically and continuously describe the multiscale and anisotropic pore structure (from nm-cm) of the shale core, and will be helpful for understanding the quality of the shale reservoir.

## Introduction

Analyses of shale cores can provide valuable information in understanding the quality of the shale reservoir and provide critical input to the completion design. Characterization of the complex pore structure in shale matrix is the foundation issue for analyses of shale cores. However, owing to the presence of multiscale pore structures and the heterogeneity at different scale, characterization of shale core is both tremendously difficult and radically different from that of conventional core samples^1,2^. A complex shale pore structure includes nano organic pores in organic matter, micro-nano inorganic pores, micro slits in inorganic mineral and micro bedding fracture in the interface of thin layers, and all these pores (slits) together play a vital role in fluid flow^3–5^. Moreover, the shale rock is made up of very thin layers, and different layer has quite different distribution of minerals which also affect the formation of bedding fractures and make the multiscale pore structure more complicated. However, many methods for modeling of porous media can construct only single-scale or single-component model and are difficult to fully classify and characterize the multiscale pore and multicomponent structure of the shale. Also, because of the high resolution required to image small scale features of interest (nm), the volumes of constructed model are inevitably small (μm^3^) when compared to the realistic shale sample (core-scale)^6^. Therefore, it is of great significance to develop new reconstructing methods to accurately reproduce the multiscale pore structure of the shale core.

At present, the methods commonly used to study the shale pore structure can be generally classified in two kinds: the physical construction method and the reconstruction algorithm. The physical construction method can directly construct real 3D digital cores through X-ray computed tomography (e.g., Micro-CT and Nano-CT) and Focused Ion Beam Scanning Electron Microscope (FIB-SEM)^6–9^. Though high-resolution imaging of the pore structure in kerogen can be obtained by FIB-SEM, FIB-SEM can only scan microscale volumes of shale. Moreover, it is still expensive and time consuming. Micro-CT and Nano-CT method can obtain much wider field of view, but they only resolve the pore throats larger than 0.7 mm and 50 nm, respectively^6,10^. Thus the physical construction method cannot satisfy the requirements of a sufficiently large, in terms of a representative elementary volume (REV), sample size and a sufficient spatial resolution.

Digital cores can also be reconstructed by 2D images through reconstruction algorithm. The reconstruction methods commonly used include the truncated Gaussian random function method, the simulated annealing method, the sequential indicator simulation method (SISIM), the multiple-point statistics (MPS) method, and various hybrid methods^11–19^. However, these techniques have never been tested for reconstructing models of shale reservoirs. Recently, Tahmasebi^20,21^ has proposed the cross-correlation–based simulation (CCSIM) method and use it to reconstruct stochastically equiprobable 3D models of shale rocks. This method can produce 3D realizations with acceptable approximation of the same properties in the 2D image. However, the reconstructed vertical morphological features are unsatisfactory^22^. The CCSIM-TSS combines the merits of both CCSIM and TSS (three step sampling method) and can reproduce more accuracy of the connectivity of the vertical direction^23^. However, it is not capable of reproducing accurately the experimental data. A hybrid method has been proposed to improve the CCSIM-TSS method by optimizing the reconstructed model based on multiple objective simulated annealing algorithm (MOSA) with experimental data as constrain conditions^24^ However, all the above methods are only suitable for constructing only single-scale or single-component digital core, and cannot be directly used for reproducing the multiscale pore structure of the shale.

Recently, there have been several studies on stochastic multiscale reconstruction of shale rocks. Gerke^25^ has developed a general solution for merging multiscale categorical spatial data into a single dataset using stochastic reconstructions with rescaled correlation functions. However, the paper only proposed this method and did not applied this method to actual 3D shale samples. Tahmasebi^21^ has develop a three-step multiresolution reconstruction method to reconstruct a shale model with a strongly bimodal distribution of the pore sizes (organic pores and few inorganic pores), and the size of the final digital core is only 10*10*10 µm^3^. As mentioned above, the complex shale pore structure includes nano organic pores, micron-nano inorganic pores, micron natural slits and micron bedding fracture, and the above method only construct two-scale and two components digital core. Also, the reconstructed result is too small to contain enough information and represent the characteristics of the shale core. Furthermore, the reconstructed result is random and its physical properties may be very different from the existing experimental data. In sum, the reconstruction of multiscale pore structure of shale is still in its infancy and needs additional study, and it is necessary to develop new methods to reconstruct more accurate model to describe the multiscale pore structure of shale core.

In this work, we present a developed core-scale reconstructing method including the multiscale and multicomponent reconstructing algorithm, the optimization algorithm and the bedding fracture constructing algorithm. To test the method, it is used to reconstruct the core-scale model of a complex shale sample from Sichuan Basin. During this process, the representative multiscale model for each layer and the bedding fractures between thin layers are reconstructed based on the proposed algorithm, and subsequently they are integrated together to obtain the final core-scale digital-experimental model. Furthermore, the morphological and flow properties of the reconstructed model are calculated and compared with the experiments. The comparison indicates that the final model honor the permeability data and pore-size distribution of the actual shale sample, which can be used for a systematic study of the fluid flow and transport in complex shale samples. Our results are expected to have practical implications in petroleum engineering.

## Results and Discussion

**Figure 1:**
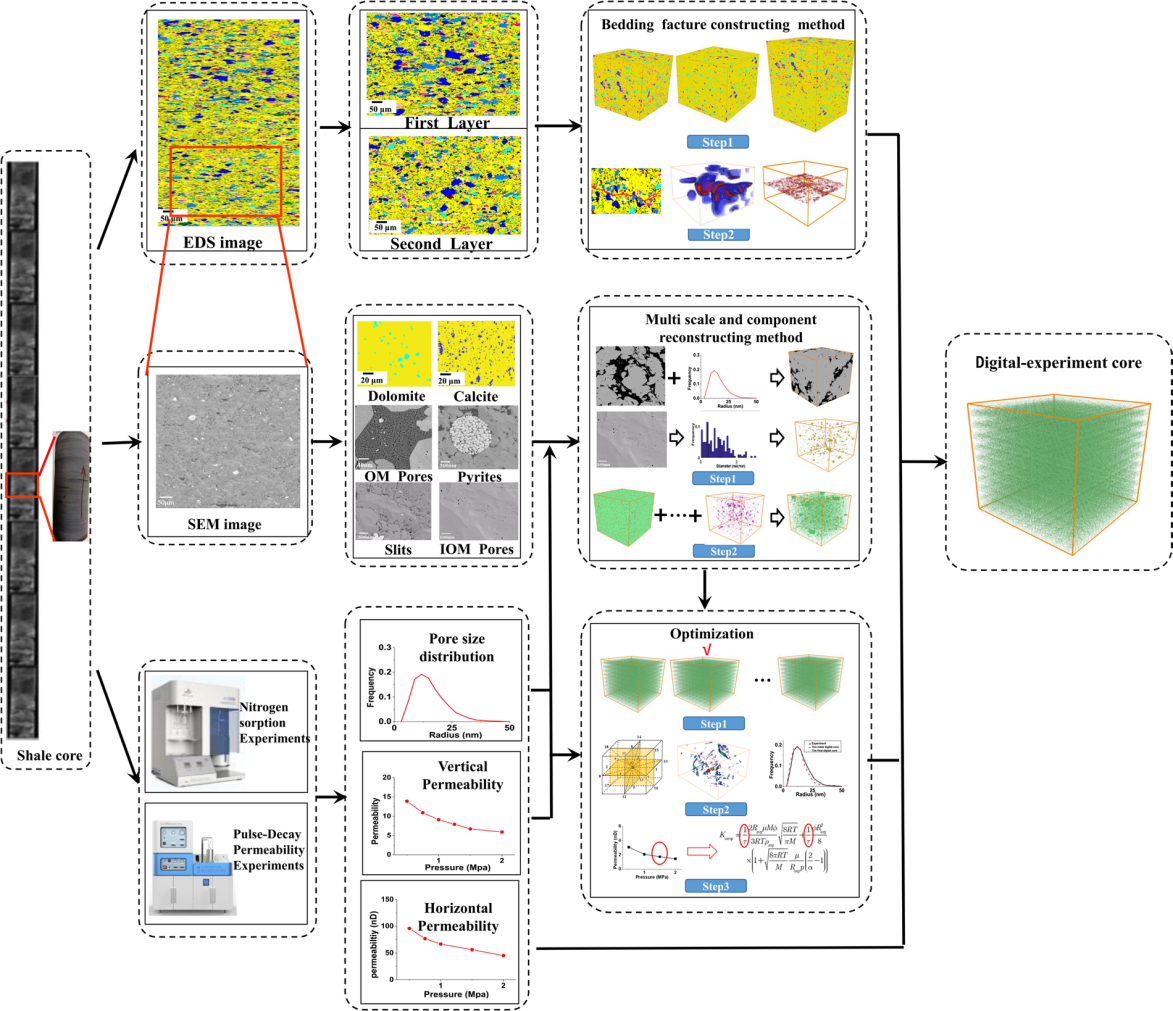
The schematic illustration of the process of reconstructing the digital-experimental model.

To test the methodology, a complex shale sample from Longmaxi Marine Shale Formation of Lower Silurian in the Sichuan Basin, are used for reconstruction. To account for the methodology, Fig. 1 plots the process of reconstructing the core-scale digital-experimental model. First, the 3D models including typical components for each thin layer are reconstructed based on 2D SEM and EDS image with experiment data(pore-size distribution) as constrain condition. Then the experimental data for such properties as flow modeling and pore size distribution are integrated to optimize the reconstructed result for each layer. Finally, the bedding fracture model and the 3D models for different layer are integrated together to obtain the final core-scale digital-experimental model, as shown in Fig. 1.

### Reconstructed 3D model for each layer

The SEM image of one thin layer in shale core, obtained by scanning electron microscopy, is shown in Fig. 1. It can be used to investigate the nanoscale pore shape and the spatial distribution of multiple pores in organic matter and typical minerals. While the EDS image, obtained by energy dispersive X-ray spectroscopy, is used to investigate the distribution of minerals in different layers of the shale core. The scanning area of SEM image, is 400 μm × 400 μm with a maximum resolution of 4.0 nm. The size of EDS map is 4000*1000 μm^2^ with a maximum resolution of 1.0 μm. By energy dispersive spectroscopy we found there are two typical thin layers in the shale core. Also, dry sample permeability of gas is measured by pulse-decay permeability measurement under 38 °C at different pressures (0.5 Mpa, 1.0 Mpa, 1.5 Mpa, 2.0 Mpa). The nitrogen adsorption method is used to characterize the pore distribution of the sample. The experiment data will be used to optimize and validate the reconstructed model. In the following we will demonstrate the process for reconstructing the representative 2D multiscale model for the first thin layer.

**Figure 2:**
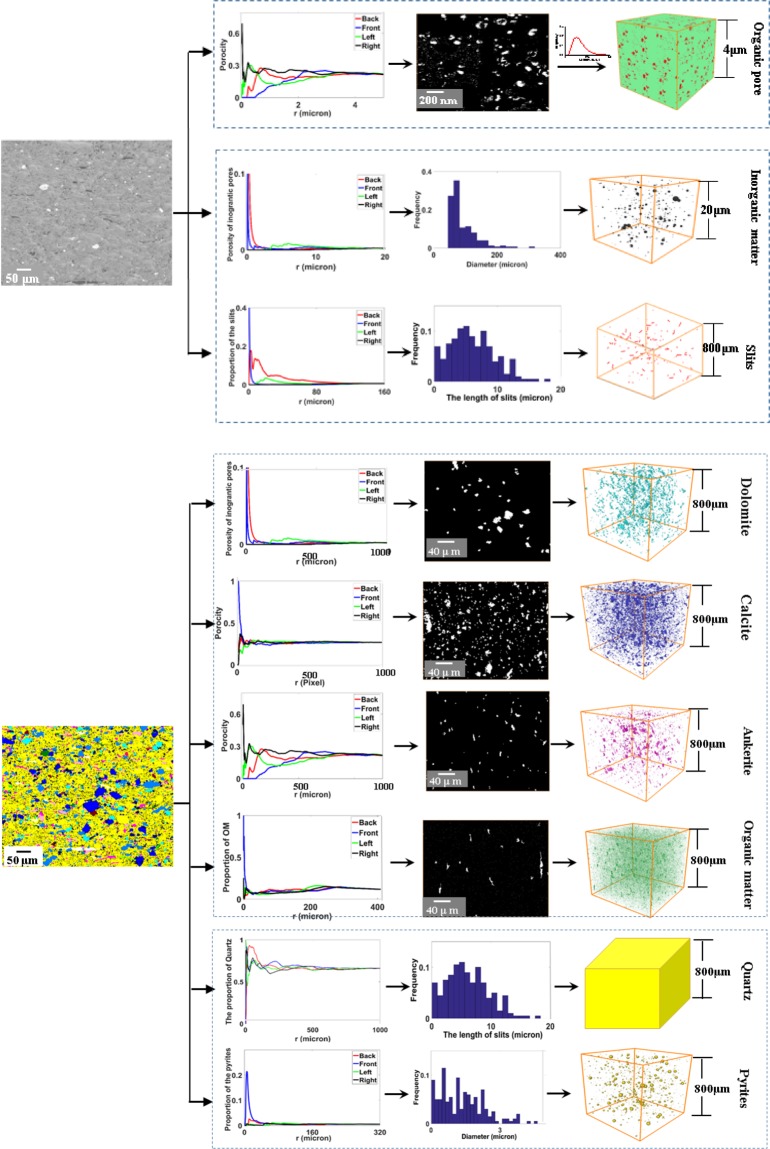
The model process of typical component in the first layer of the shale sample.

The typical components, such as organic pores, inorganic pores, slits, pyrites, organic matter (OM), dolomite, calcite, ankerite and quartz, are extracted from the SEM and EDS image at different resolutions. To ensure that each image can represent the characteristics of the corresponding component, the RES (the representative elementary surface) for each component is calculated, as shown in Fig. 2. We choose four different initial points (four corners of an image), expand the target area starting from the source points (1 pixel on the edge) in the 2D image and calculate the proportion of each component in the sub-square. Inspecting Fig. 2, we can find that the lengths of the edge of the RES for organic pores, inorganic pores, slits, pyrites, organic matter (OM), dolomite, calcite, ankerite and quartz are approximately 4 μm, 15 μm, 120 μm, 200 μm, 320 μm, 800 μm, 350 μm and 800 μm, respectively.

Then the 3D representative model for the first layer are reconstructed by the proposed multi scale and component approach as follow: First, typical components in the shale are reconstructed. Organic matter has abundant nanopores which connect the macroscale pores, and it is very important in representing an actual interconnected pore network. To accurately reproduce the connectivity of the nanopore structure, the 3D models of organic matter and organic pores are reconstructed by the CCSIM-TSS algorithm based on the representative 2D image, as shown in Fig. 2. The volumes of the realizations are 800*800*800 μm^3^ and 4*4*4 μm^3^, and the resolution are 100 nm and 4 nm, respectively. Also, the 3D model for dolomite, calcite and ankerite are reconstructed by the CCSIM-TSS method, and their volumes are 800*800*800 μm^3^. As they manifest, the global structures in the 2D image are reproduced. Then, the 3D models for the inorganic pores, pyrites and slits are constructed by the statistical analysis algorithm. The sizes of the 3D models for the inorganic pores, pyrites and slits are 20*20*20 μm^3^, 800*800*800 μm^3^ and 800*800*800 μm^3^, and the resolutions are 10 nm, 1000 nm, and 1000 nm, respectively. The size of the 3D models for pyrites and slits is bigger than that of the RES image because it is convenient for superposing them with the 3D models for organic matter.

**Figure 3:**
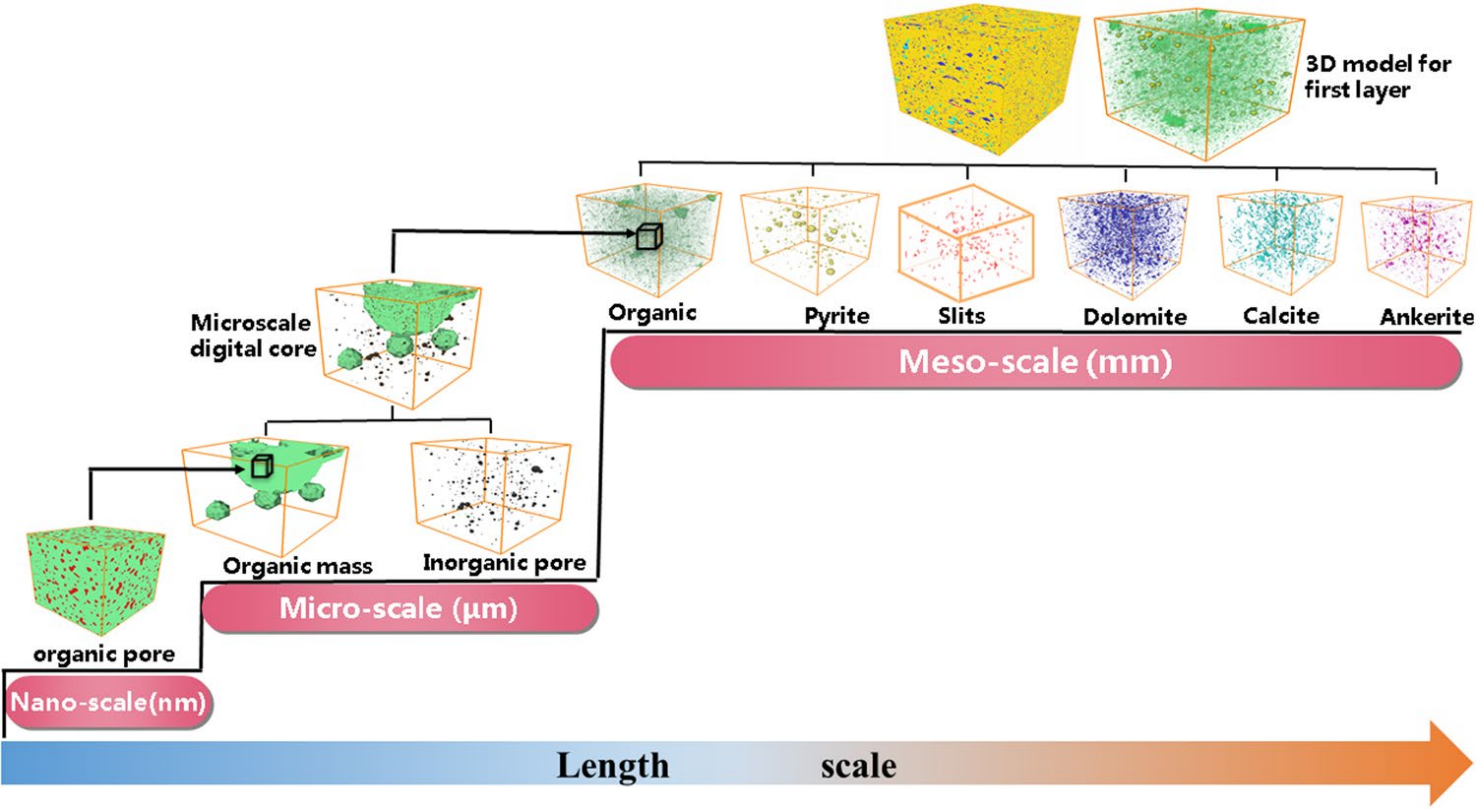
The process of merging various scale digital cores together.

Second, the models for the typical components are integrated (see Fig. 3). Up to now, nine different models for typical components in the first layer are obtained, and they represent the shale from microscale and nanoscale formations, respectively. At first, the kerogen solids (green) and organic pores (red) on the organic pore image (4*4*4 µm^3^) is merged with the organic matter model (800*800*800 µm^3^) and the inorganic pores model (20*20*20 µm^3^). It should be noted that the nanoscale image of organic pores is only filled in the organic matter in the organic matter model and the inorganic pores is only filled in the skeleton in the organic matter model. Especially, the models for inorganic pores for different mineral are only filled in the corresponding mineral region. Then the 3D models with organic matter and pores in the OM and IOM are integrated with the models for the pyrites, slits, dolomite, calcite, ankerite and quartz together based on the multiscale superposition algorithm. The final multiscale model (800*800*800 µm^3^) for the first layer is plotted in Fig. 3, and it can be found that it has multiscale pore structures and multi components.

**Figure 4:**
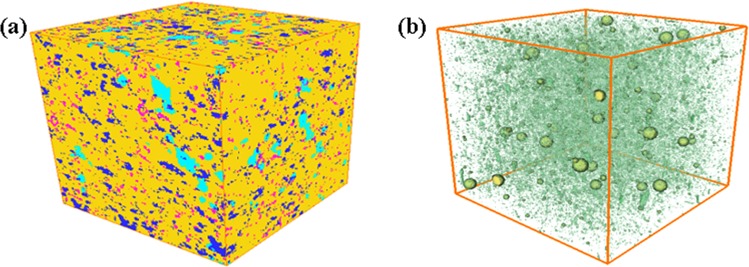
The reconstructed 3D model in the second layer of the shale sample (1000*1000*1000 µm^3^). (**a**) 3D model containing several typical minerals; (**b**) the corresponding 3D model (1000*1000*1000 µm^3^) only including OM, pyrites and multiscale pores (slits).

Like the reconstruction of the model for the first layer, the 3D representative model for the second layer is generated, as shown in Fig. 4, and its size is 1000*1000*1000 µm^3^. To examine the pore structure more clearly, we also plot the corresponding 3D model only including OM, pyrites and multiscale pores (slits). It can be seen from the models that the mineral structure of the second layer is quite different from that of the first layer.

### Optimization for the 3D model in each layer

Although the multiscale and multicomponent algorithm can reproduce models with the high accuracy of statistical properties, the reconstructed models are random and their physical properties may be different from the experimental data. Thus in the following we will use the experiment data as constrain condition to optimize the reconstructed multiscale models.

**Figure 5:**
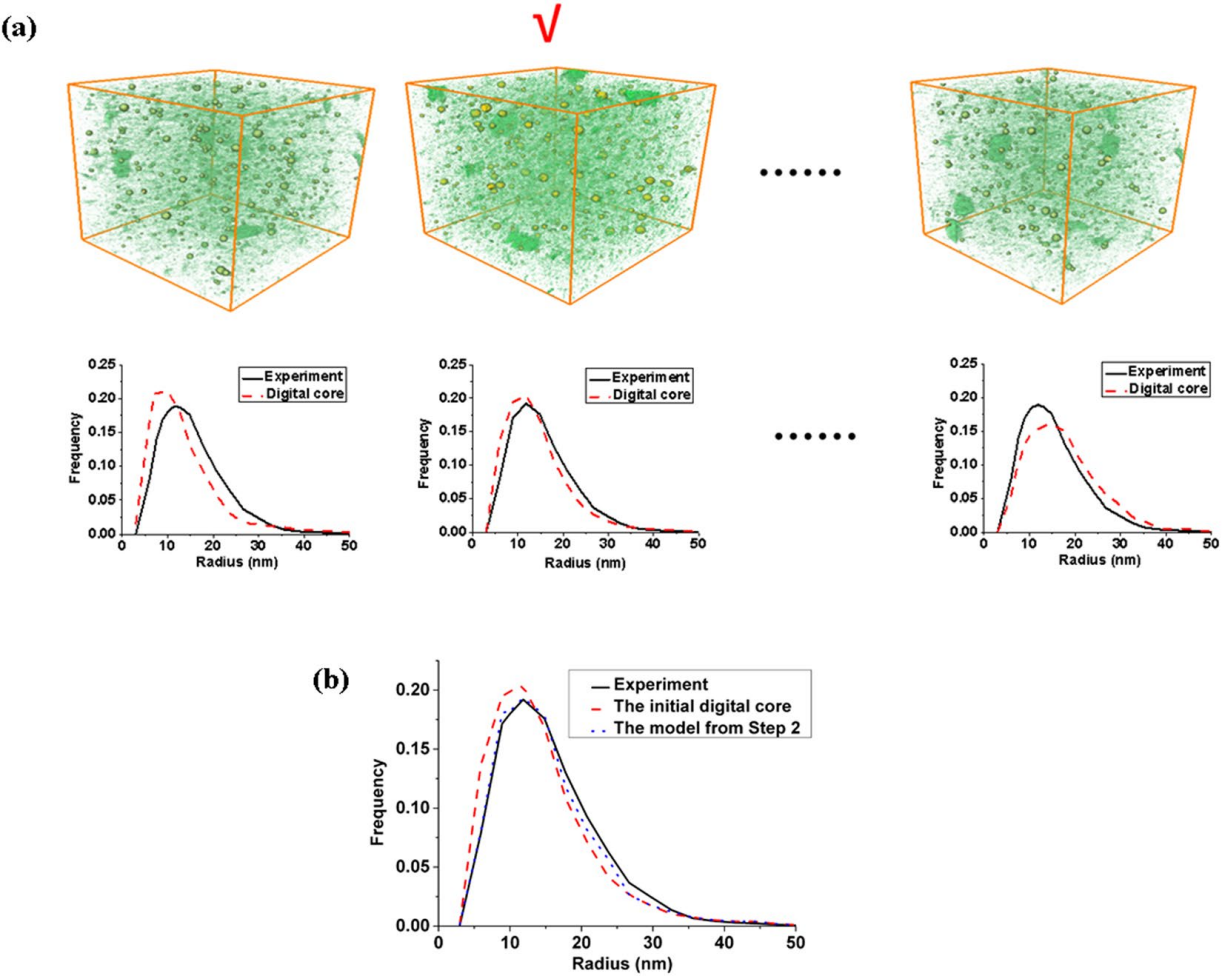
Selecting the most accurate pore size distribution. In this case the most accurate model for the first layer is the second (**a**). Comparison of the pore size distribution of the final 3D model for the first layer obtained by the optimization method, before optimization and the experiment data (**b**).

First, 10 multiscale realizations for each layer are generated, and the pore size distribution of each realization is calculated. The one that most approach the experiment data is chose, as shown in Fig. 5a. Second, the model is optimized to minimize the difference between it and the experiment data. The result in Fig. 5b indicates that the optimized model obtained by the Step 2 reproduces the pore-throat size distribution of the real samples very well. Third, the apparent permeability of the 3D model in the above step is calculated by the multiscale gas transport simulation method (see Supplementary File). The tortuosity of the inorganic pores is determined by the vertical permeability obtained from experiment at 1.5 MPa. It can be obtained that the tortuosity of the inorganic pores for 3D model of the first and second layer are 2.6 and 2.1.

### The final core-scale digital-experimental model

**Figure 6:**
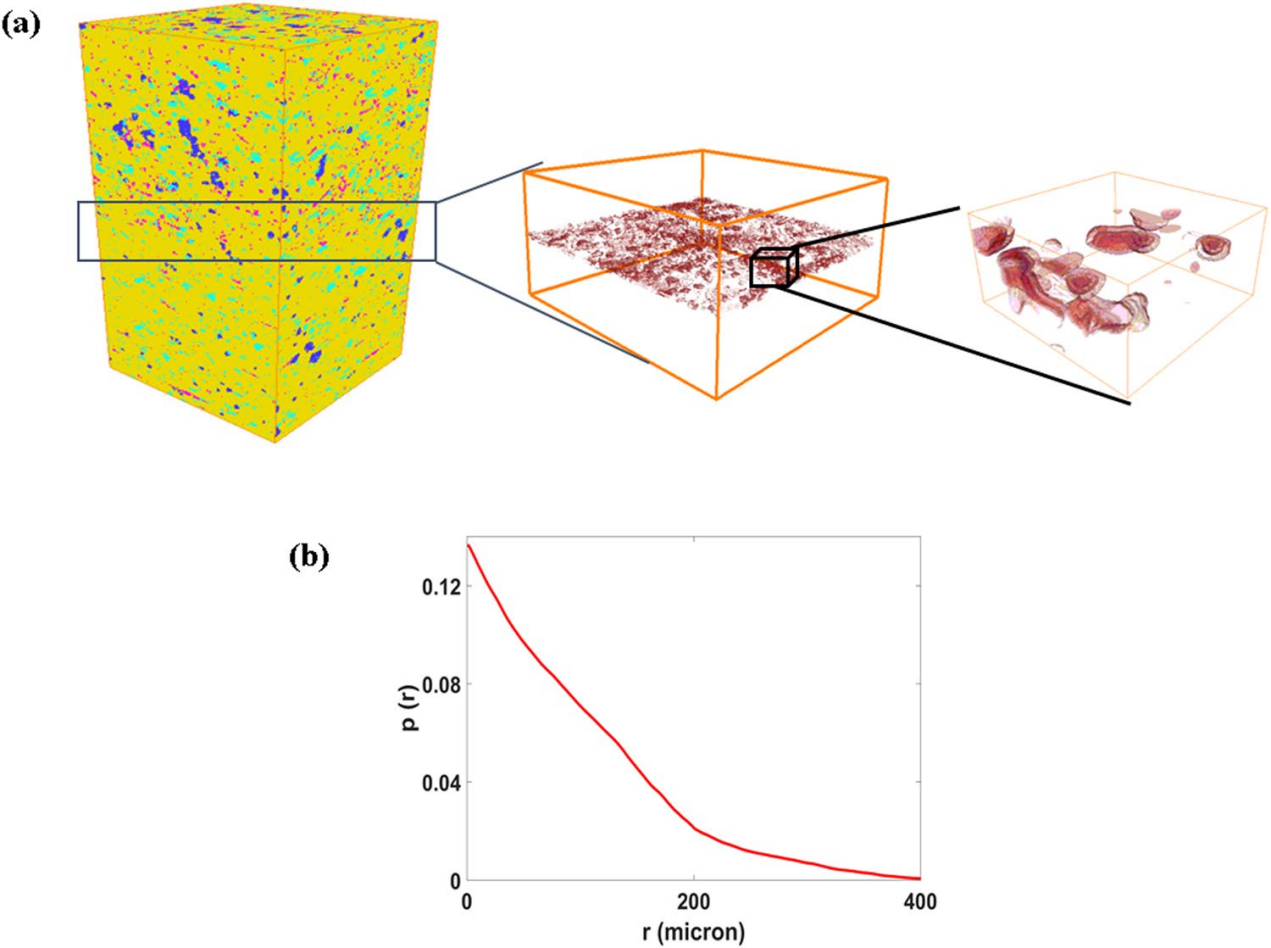
Comparison of the pore size distribution of the final result obtained by the optimization method, before optimization and the experiment(**a**); The connectivity of the bedding fractures.

In the following we will construct the digital-experimental core with multi layers. First, based on the bedding fracture constructing method and the mineral distribution in the interface of two layers, the bedding fracture are constructed, as shown in Fig. 6a. It can be found that the bedding fractures are curved surface. Furthermore, we calculate the multiple point connectivity of the bedding fractures. Figure 6b shows that the connectivity of the bedding fractures is good, and it plays a very important role in the flow of the shale gas. For more detail information of the bedding fracture, please see the Supplementary File.

**Figure 7:**
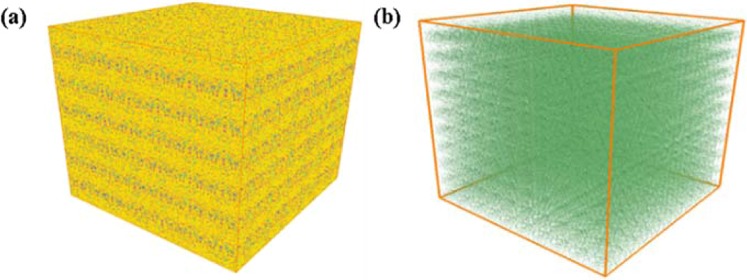
The final digital-experimental 3D model (2.0*2.0*2.0 cm^3^) (**a**); The corresponding 3D model only including OM, pyrites and multiscale pores (slits) (2.0*2.0*2.0 cm^3^) (**b**).

Similar to the above the method, the 3D representative multiscale models of each layer are integrated together to obtain the final core scale 3D model. Considering there are only two typical layers in our shale core sample, the 3D representative models for each layer are alternately superposed together. Similar to the inorganic pores, the horizontal permeability at the 1.5 MPa is used as constrain condition to optimize the 3D model and the tortuosity of the bedding fracture is 1.5. The final digital-experimental 3D model are shown in Fig. 7. The size of the model is 2.0*2.0*2.0 cm^3^ and the resolution is 4 nm. In the same way, to examine the pore structure more clearly, we also plot the corresponding 3D model only including OM and pores (Fig. 7b). It can be seen from the figure that the model are layered.

### Validation

**Figure 8:**
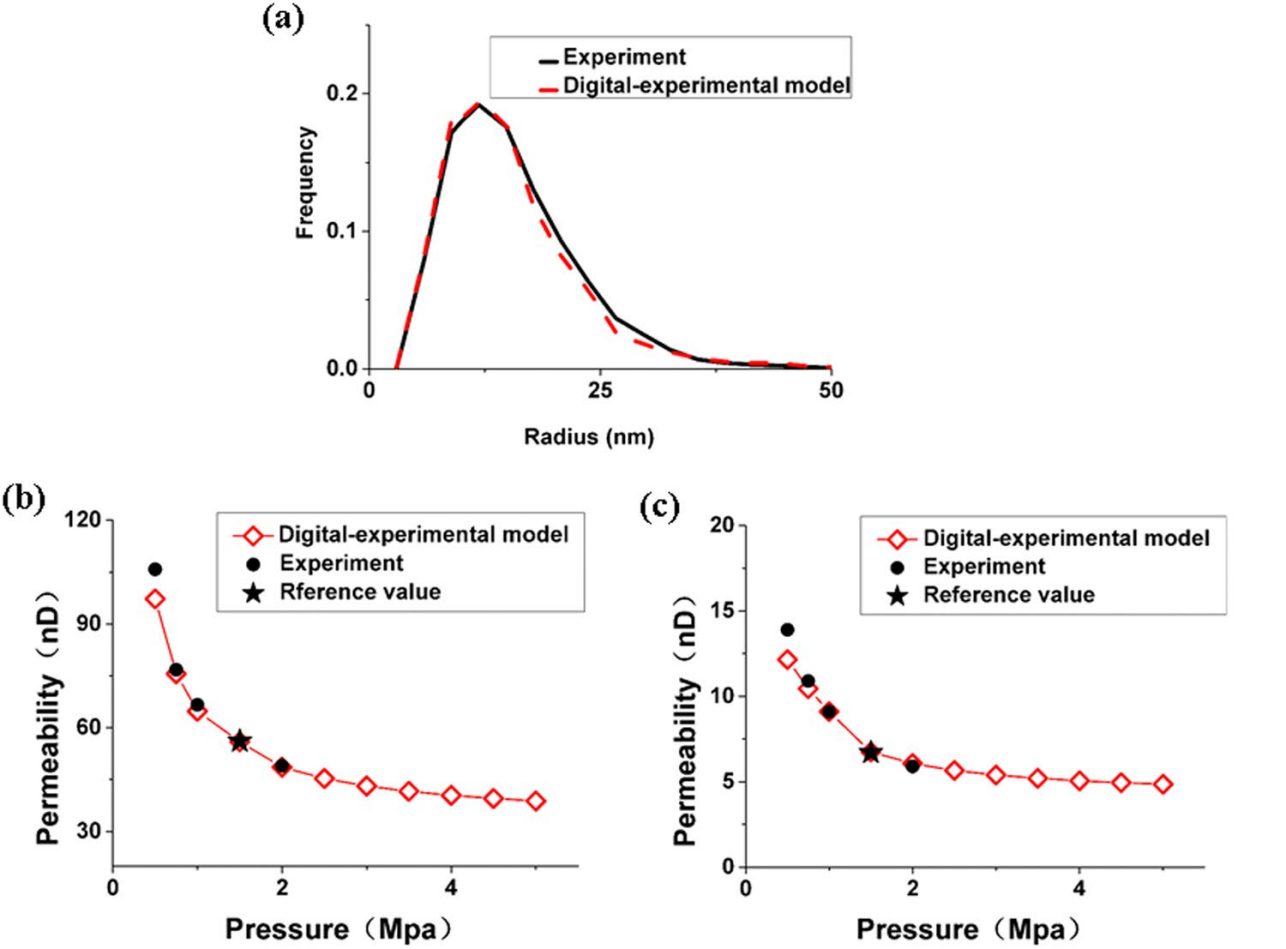
Comparison of the pore size distribution (**a**) and the permeability in the horizontal (**b**) and vertical direction (**c**) obtained from experiment and digital-experiment cores of shale sample.

Finally, we present a comparison between the pore size distribution and the apparent permeability of the final digital-experiment model and the actual shale core to validate it, as shown in Fig. 8. Figure 8a shows that the pore size distribution of the digital-experiment cores and experiment data of the actual shale rock agrees very well. Figure 8b,c demonstrates that the values of apparent permeability from the simulations agree well with the results from experiments at the average pressure of 0.5 MPa, 0.75 MPa, 1.0 MPa and 2.0 Mpa. The good agreement of the apparent permeability validated the reconstructed digital-experimental core.

## Conclusions

Constructed more realistic core-scale 3D models with sufficient spatial resolution is of great significance for exploration of shale reservoirs. In this paper, the core-scaling reconstructing method, including the multiscale and multicomponent reconstructing algorithm, the optimization algorithm and the bedding-fracture constructing algorithm, is used to reconstruct the digital-experimental model considering the multiscale pores. By using multiscale and multicomponent reconstructing algorithm, the representative multiscale model for each layer is successfully built based on the 2D SEM and EDS image. With the pore size distribution and vertical permeability obtained from experiment as constrain condition, the representative multiscale model are adjusted by the optimization algorithm to accurately describe the 3D structures of nano organic pores, micro-nano inorganic pores, micro slits and several typical minerals for each layer. Finally, the bedding fracture constructing algorithm guarantees an accurate reproduction of the complex bedding fracture distribution and the representative multiscale models for different layers are integrated together to obtain the final core-scale digital-experiment model. The digital-experiment model can systematically and continuously describe the multiscale and anisotropic pore structure in organic-rich shale core, and will be helpful for understanding the quality of the shale reservoir and provide critical input to the completion design.

The method proposed in this paper generates realization of shale samples that match the measured permeability and the pore size distribution only based on a large area 2D SEM image, EDS image and experiment data without using full 3D imaging. It can be used as a tool for reducing the cost and time in the studies of shale.

## Methodology

Due to the wide pore size distribution ranging from nanometer to micrometer and the multiple layers in shale core, it is very difficult to consider different pores and components simultaneously and the combination of various algorithms is essential to obtain the “global 3D model” of the shale. The main idea of the core-scale reconstructing method proposed in our paper is to characterize the 3D structure of different pores and components of shale with different techniques and then integrate them together. The proposed methodology in this paper consists of three algorithms used sequentially to produce high-quality 3D model.

### The multiscale and multicomponent reconstructing algorithm

**Figure 9:**
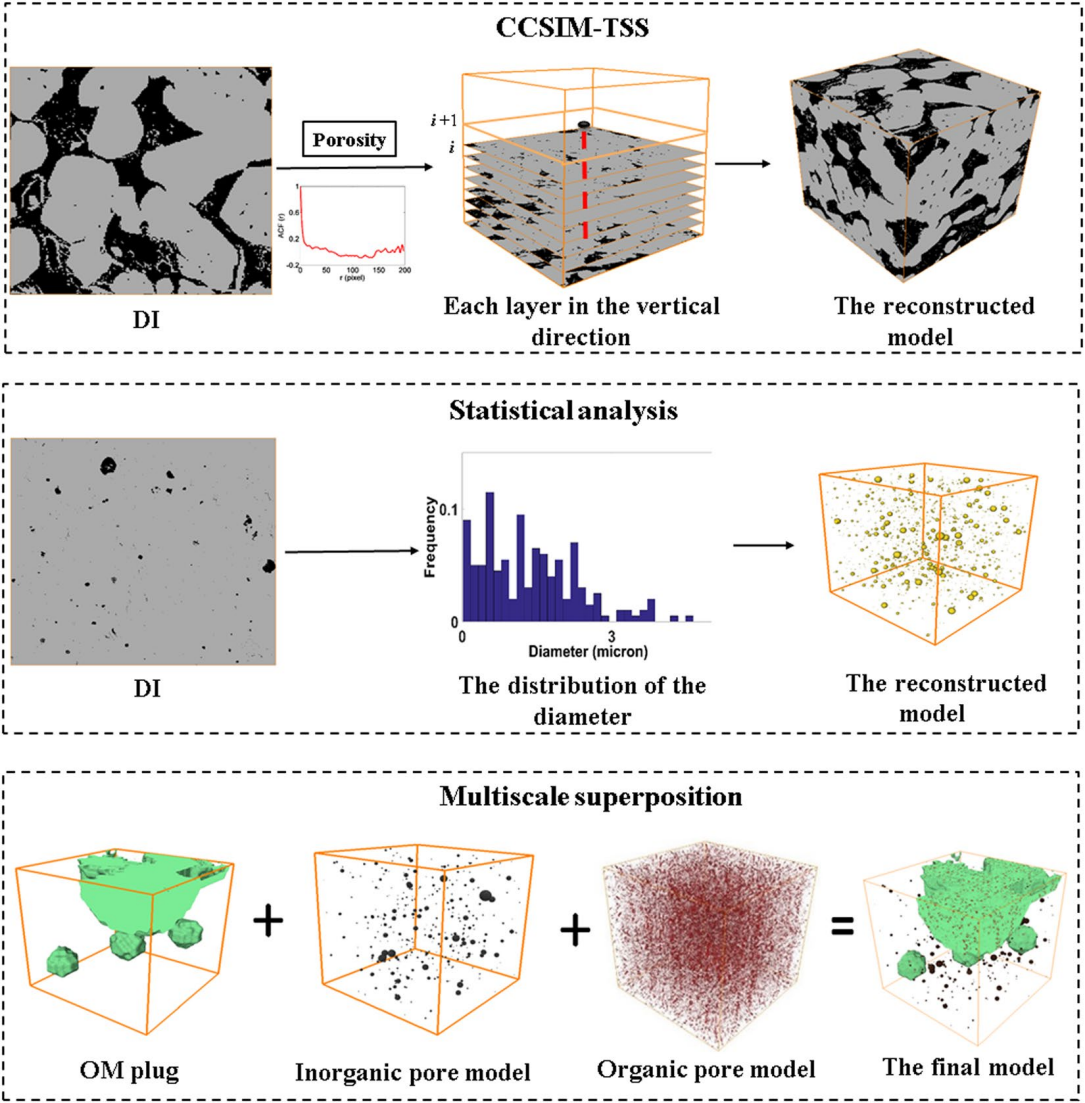
The schematic illustration of the multiscale and multicomponent reconstructing algorithm.

This algorithm utilizes the cross correlation based simulation- three step sampling method (CCSIM-TSS) to produce an ensemble of realizations for inhomogeneous porous media^23^. In our work, the CCSIM-TSS method is used to reproduce the morphological features of some components with complex structure in the shale. The steps of the CCSIM-TSS method is arranged as follows (see Fig. 9). First, a representative 2D image, named as DI, is selected as the digital image. Second, the image is set as the first layer at the bottom part of the reconstructed 3D model, and the other four frames (left, right, front, and back) are reconstructed. Third, the internal structure is generated layer by layer in the vertical direction. The multiple-point connectivity probability function of the DI is calculated. Then, if the k + 1 layer is reconstructed currently, the k layer is successively scanned by 5 × 5 and 3 × 3 sampling templates. If the points of the template, are entirely pore (grain), the central node is marked as a sampling point. Based on the multiple-point connectivity probability function, the sampling points in the remaining area are selected. The sampling points are used as condition constrain when reconstructing the k + 1 layer, thus the continuity and variability between adjacent layers can be controlled directly. Finally, the all the layers are stacked together to obtain the reconstructed 3D model.

The multiscale and multicomponent reconstructing algorithm utilizes the statistical analysis algorithm to generate the 3D models of some components in the shale, which are scattered in the shale matrix and their connectivity are not very good. The procedure of the statistical analysis algorithm is: (1) the diameter distribution of each component (pyrites and inorganic pores) is calculated based on the global SEM image at proper resolution; (2) we use the statistics to construct random spheres whose radius follow the distribution of the diameter, and the spheres are distributed randomly in the 3D image (digital core), as shown in Fig. 9.

In order to capture the pores at various scales, the multiscale superposition algorithm are utilized to integrate the 3D models with different physical size and resolution together. The procedure is: (1) the macropore digital core with low resolution is refined into an image with high resolution using a cubic spline interpolation technique. During this step, each voxel in macropore digital core is refining into i*i*i voxels (i is the resolution ratio between the low resolution pore digital core and the high resolution one). (2) We stack j*j*j nanopore digital cores with high resolution up to obtain a composition image with the same size of the macropore digital core. (3) The 3D model for different component is superposed as follows:

1 $$\begin{array}{ccc}{\rm{\Omega }} & = & {{\rm{\Omega }}}_{1}+{{\rm{\Omega }}}_{2}+{{\rm{\Omega }}}_{3}+{{\rm{\Omega }}}_{4}+{{\rm{\Omega }}}_{5}+{{\rm{\Omega }}}_{6}+{{\rm{\Omega }}}_{7}+{{\rm{\Omega }}}_{8}\\ {{\rm{\Omega }}}^{skeleton} & = & {{\rm{\Omega }}}_{1}^{skeleton}+{{\rm{\Omega }}}_{3}^{skeleton}+{{\rm{\Omega }}}_{4}^{skeleton}+{{\rm{\Omega }}}_{5}^{skeleton}+{{\rm{\Omega }}}_{6}^{skeleton}+{{\rm{\Omega }}}_{7}^{skeleton}\\  &  & +\,{{\rm{\Omega }}}_{8}^{skeleton}\\ {{\rm{\Omega }}}^{OM} & = & {{\rm{\Omega }}}_{1}^{OM}+{{\rm{\Omega }}}_{3}^{skeleton}+{{\rm{\Omega }}}_{4}^{skeleton}+{{\rm{\Omega }}}_{5}^{skeleton}+{{\rm{\Omega }}}_{6}^{skeleton}+{{\rm{\Omega }}}_{7}^{skeleton}\\  &  & +\,{{\rm{\Omega }}}_{8}^{skeleton}\\ {{\rm{\Omega }}}^{Pyrite} & = & {{\rm{\Omega }}}_{1}^{skeleton}+{{\rm{\Omega }}}_{3}^{Pyrite}+{{\rm{\Omega }}}_{4}^{skeleton}+{{\rm{\Omega }}}_{5}^{skeleton}+{{\rm{\Omega }}}_{6}^{skeleton}+{{\rm{\Omega }}}_{7}^{skeleton}\\  &  & +\,{{\rm{\Omega }}}_{8}^{skeleton}\\ {{\rm{\Omega }}}^{dolomite} & = & {{\rm{\Omega }}}_{1}^{skeleton}+{{\rm{\Omega }}}_{3}^{skeleton}+{{\rm{\Omega }}}_{4}^{skeleton}+{{\rm{\Omega }}}_{5}^{skeleton}+{{\rm{\Omega }}}_{6}^{dolomite}+{{\rm{\Omega }}}_{7}^{skeleton}\\  &  & +\,{{\rm{\Omega }}}_{8}^{skeleton}\\ {{\rm{\Omega }}}^{calcite} & = & {{\rm{\Omega }}}_{1}^{skeleton}+{{\rm{\Omega }}}_{3}^{skeleton}+{{\rm{\Omega }}}_{4}^{skeleton}+{{\rm{\Omega }}}_{5}^{skeleton}+{{\rm{\Omega }}}_{6}^{skeleton}+{{\rm{\Omega }}}_{7}^{calcite}\\  &  & +\,{{\rm{\Omega }}}_{8}^{skeleton}\\ {{\rm{\Omega }}}^{ankerite} & = & {{\rm{\Omega }}}_{1}^{skeleton}+{{\rm{\Omega }}}_{3}^{skeleton}+{{\rm{W}}}_{4}^{skeleton}+{{\rm{\Omega }}}_{5}^{skeleton}+{{\rm{\Omega }}}_{6}^{skeleton}+{{\rm{\Omega }}}_{7}^{skeleton}\\  &  & +\,{{\rm{\Omega }}}_{8}^{ankerite}\\ {{\rm{\Omega }}}^{OMpore} & = & {{\rm{\Omega }}}_{1}^{OM}+{{\rm{\Omega }}}_{2}^{{\rm{O}}{\rm{M}}pore}\\ {{\rm{\Omega }}}^{IOMpore} & = & {{\rm{\Omega }}}_{1}^{skeleton}+{{\rm{\Omega }}}_{3}^{skeleton}+{{\rm{\Omega }}}_{4}+{{\rm{\Omega }}}_{5}^{IOM\,pore}+{{\rm{\Omega }}}_{6}+{{\rm{\Omega }}}_{7}+{{\rm{\Omega }}}_{8}\\ {{\rm{\Omega }}}^{slit} & = & {{\rm{\Omega }}}_{1}^{skeleton}+{{\rm{\Omega }}}_{3}^{skeleton}+{{\rm{\Omega }}}_{4}^{slit}+{{\rm{\Omega }}}_{5}+{{\rm{\Omega }}}_{6}+{{\rm{\Omega }}}_{7}+{{\rm{\Omega }}}_{8}\end{array}$$

where Ω, Ω_1_, Ω_2_, Ω_3_, Ω_4_, Ω_5_, Ω_6_, Ω_7_, Ω_8_, Ω_9_ indicates the final superposed shale multiscale digital core, the organic matter digital core, the organic pores digital core, the pyrite digital core, the slits network, the inorganic pores digital core, the dolomite digital core, the calcite digital core, the ankerite digital core and the quartz digital rock, respectively. For the superposed shale multiscale digital core, Ω^*skeleton*^, Ω^*pyrite*^, Ω^*organic pore*^, Ω^*inorganic pore*^, Ω^*slit*^, Ω^*om*^, Ω^dolomite^, Ω^calcite^ and Ω^anlerite^ represent the skeleton, the pyrites, the organic pores, the inorganic pores, the slits, organic matter (OM), dolomite, calcite, ankerite, and their values are 0, 1, 2, 3, 4, 5, 6, 7 and 8. Figure 9 presents an example of the superposition of the organic matter digital rock, the organic pores digital rock, the inorganic pores digital core. It should point out that this multiscale superposition algorithm applies to the cases that the difference of resolutions is not too big or the heterogeneities are not too strong.

### The optimization algorithm

**Figure 10:**
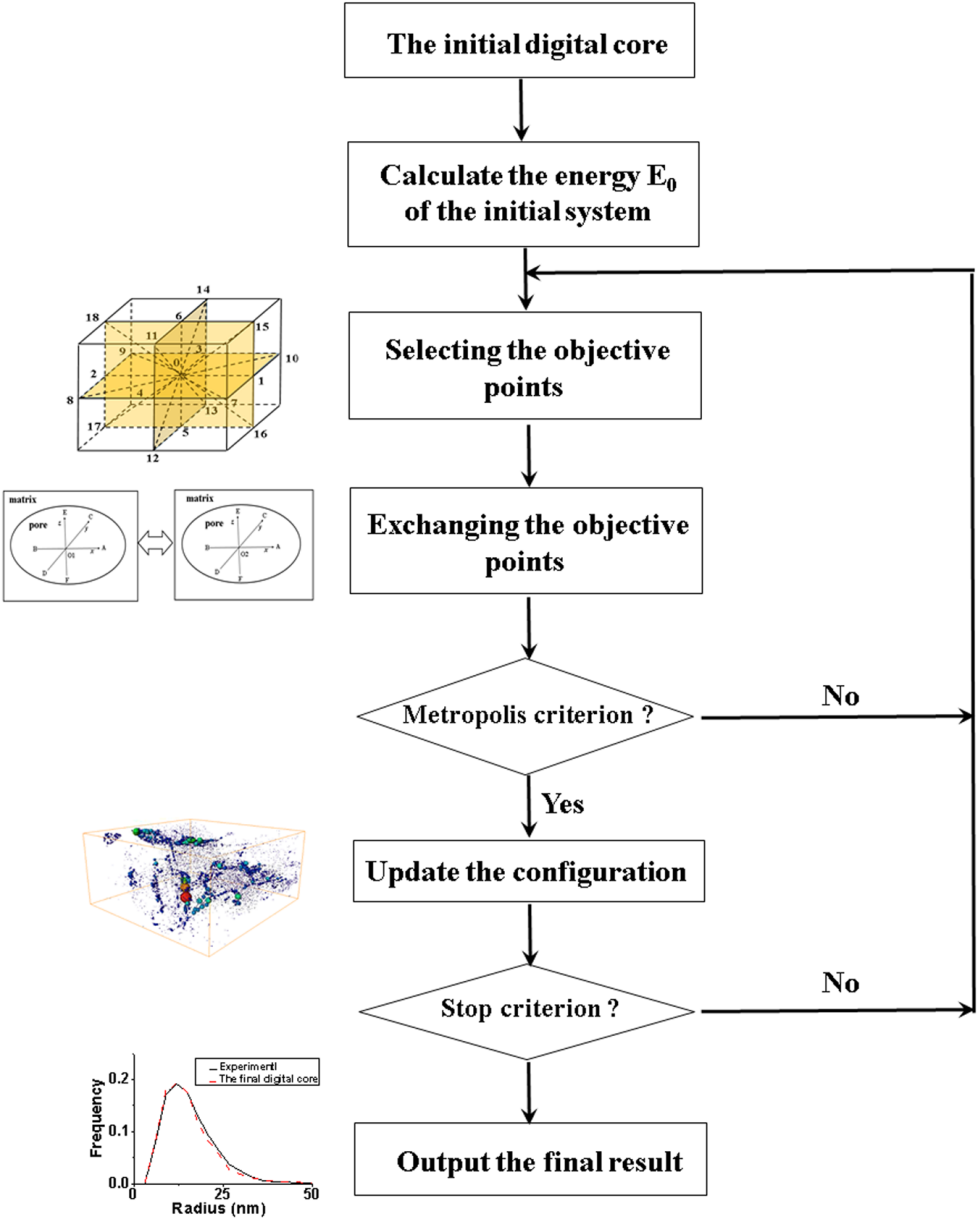
The flow chart of the step 2 in optimization algorithm.

The optimization algorithm consists of three steps, and each step relies on the model developed in the previous steps and improves it. (1) This step chooses the reconstructed model whose physical properties is most close to the experiment data (pore-size distribution) from the realizations reconstructed by the statistic method. It should be pointed out that the AB (axis & ball) algorithm is used to extract the pore networks from the reconstructed model and the pore-size distribution is calculated based on the pore networks^26^. (2) The complete pore-size distribution of the reconstructed model is sometimes different from the experiment data by Step 1. Thus, the model in Step 1 is further improved using an iterative scheme to minimize the difference and reproduce model that is even more similar to the real sample, as shown in Fig. 10. To this end, the 3D model reconstructed in Step 1 is further optimized based on simulated annealing algorithm (SA) with the experiment data (pore-size distribution) as the objective function^24^. We design the search scheme based on D3Q19model, select the boundary point of pore and the matrix, and proposes the generation solutions of the new system by exchanging the boundary points of two pores. Then the Metropolis criterion is used to determine whether the new system is accepted. The iteration process continues until the difference between the reconstructed model and the experiment becomes lower than a specified value. The flow chart of the optimization algorithm is shown in Fig. 10; (3) The continuity of the inorganic pores of the multiscale model in Step 2 is random and the apparent permeability may be different from the experiment data. Here we assume that the tortuosity of the inorganic pores in inorganic matrix is the same, and it is determined by the apparent permeability obtained from the experiment data. The apparent permeability of the multiscale model is calculated by the multiscale gas transport simulation method.

### The bedding fracture constructing method

**Figure 11:**
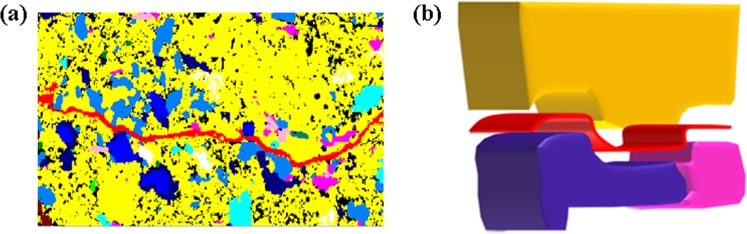
The 2D EDS image of bedding fracture (**a**) and the formation of 3D bedding fracture (red curved surface) (**b**).

Inspecting the 2D EDS image, we can find that the bedding fracture distributes mainly in the interface of two different minerals of the two layers, as shown in Fig. 11a. Thus, the bedding fracture constructing method construct the 3D bedding fracture network as follows: (1) during superposing two 3D representative models (names as model A and model B) of different thin layers together, we search the contact surface of two mineral grains in the interface of model A and model B. (2) If these two mineral grains belong to different models and have different kinds of minerals, we assume that the bedding fracture are formed in their contact surface. For example, as shown in Fig. 11b, there are three different mineral grains. The yellow mineral grain belong to model A, while the blue and pink mineral grains belong to model B. Thus there is a bedding fracture in the contact surface between the yellow and blue (pink) mineral grains. However, there are no bedding fracture between the blue and pink mineral grains, because they both belong to model B. According to the above method, we can obtain the bedding fracture network between two different layers.

## Supplementary information

Supplementary File
