# Supplementary material for: A core-scale reconstructing method for shale
Source: Sci Rep. 2019 Mar 13;9:4364. doi: 10.1038/s41598-019-39442-5 (PMC6416323; doi:10.1038/s41598-019-39442-5)
Supplement: Supplementary file 1 — Supplementary File [file 41598_2019_39442_MOESM1_ESM.docx]

**Supplementary Materials for**

**A core-scale reconstructing method for shale**

**Authors:**

**Lili Ji^1^ Mian Lin^1,2^,* Gaohui Cao^1,2^ Wenbin Jiang^1^**

**Affiliations:**

**^1^***Key Laboratory for Mechanics in Fluid Solid Coupling Systems, Institute of Mechanics, Chinese Academy of Sciences, Beijing, 100190, China*

**^2^** *University of Chinese Academy of Sciences, Beijing, 100190, China*

**This file includes:**

Supplementary Text

Figs. S1

Figs. S2

Figs. S3

Supplementary Reference List

**Supplementary Text**

**Pulse-decay permeability measurement for Organic-rich shale** Dry sample permeability of gas is measured by pulse-decay permeability measurement under 38 °C. The tri-axial permeability rig used in this work is shown schematically in Fig. S1.

The pressure decay curve can be modelled as:

$\frac{p_{u}\text{-}p_{d}}{p_{u,0}-p_{d,0}}=e^{-\alpha t}$ (S1)

where $p_{u}$ and $p_{d}$ are the pressure of the upstream and downstream cylinders, respectively. $p_{u,0}$ and $p_{d,0}$ are the pressure of the upstream and downstream cylinders at the initial stage, $t$ is time, and $\alpha$ can be expressed as (Pan et al, 2015):

$\alpha=\frac{K_{m}A(p_{u,0}+p_{d,0})}{2\mu L}(\frac{1}{V_{u}}+\frac{1}{V_{d}})$ (S2)

where $K_{m}$ is the permeability of shale matrix, $L$ is the sample length, $A$ is the sample cross-section area, $\mu$ is gas viscosity, and $V_{u}$ and $V_{d}$ are the volume of the upstream and downstream cylinders, respectively.

**Figure. S1 Schematic of the tri-axial apparatus for pulse-decay permeability measurement.**

**The multiscale gas transport simulation.** Based on the multiscale digital core, a workflow can be constructed specifically to calculate the apparent permeability k. To account for the characteristics of different scales, the proposed workflow consists of two levels^2^. At level I, the permeability distributions for OM and IOM are determined, respectively. At level II, the finite volume method are used to simulate the apparent permeability based on the multiscale digital core.

In the OM, the model proposed by Jiang^3^ are used to calculate the permeability:

(S3)

where $N$ is the total number of throats and$r_{i}$ is the radius of throat $i$, is the pore surface fractal dimension, tortuosity , and the flowing porosity. *M* is the gas molar mass, *R* is the gas constant, *T* is the temperature. *μ* is the viscosity of gas, $p$is the pressure of the gas..

In the IOM, the permeability for inorganic pores $K_{iomp}$ is estimated with the theoretical model proposed by Darabi et al^4^. Especially, here we assume that the D_f_ is 2, and the tortuosity of inorganic pores $\tau$will be determined by the permeability obtained from the experiment.

(S4)

where is the porosity, tortuosity, average pore radius of the inorganic pores.

In the IOM, the permeability for the slits is estimated with the theoretical model as^28^

(S5)

where h is the width of the slits, is the porosity and tortuosity of the slits.

Based on the above models of shale solid, numerical simulations are performed with the finite volume method. It should be pointed that the domain size of the shale solid should be larger than the REV for the spatial distribution feature of OM and IOM. The numerical tests are carried out under various average pressures.

**The** **multiscale superposition algorithm**. To account for the effect of the cubic spline interpolation technique in the multiscale superposition algorithm, we plot one part of the reconstructed result of the organic matter, as shown in Fig. S2. To see more clearly, one slice of each 3D model are demonstrated. It can been obtained that before using cubic spline interpolation technique, the boundary of the reconstructed result is sharp and not smooth. While after using cubic spline interpolation technique, the above problem can be solved.


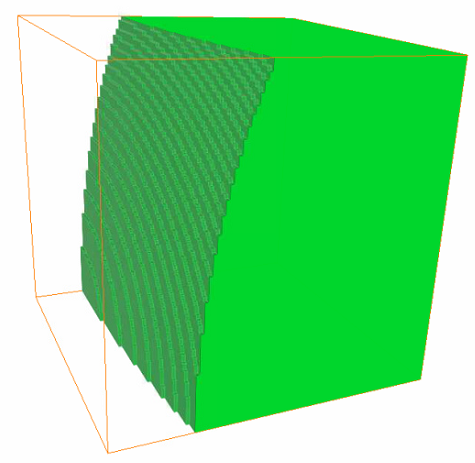

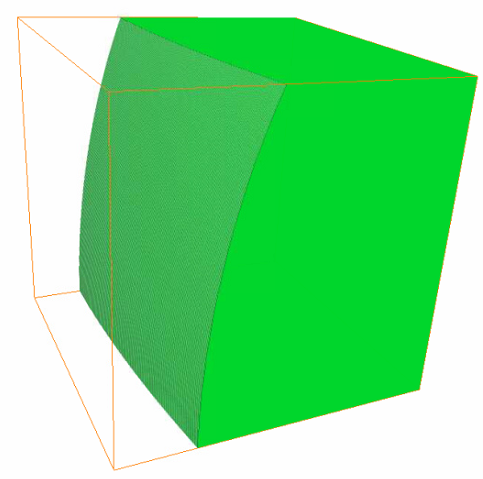


1. **(b)**


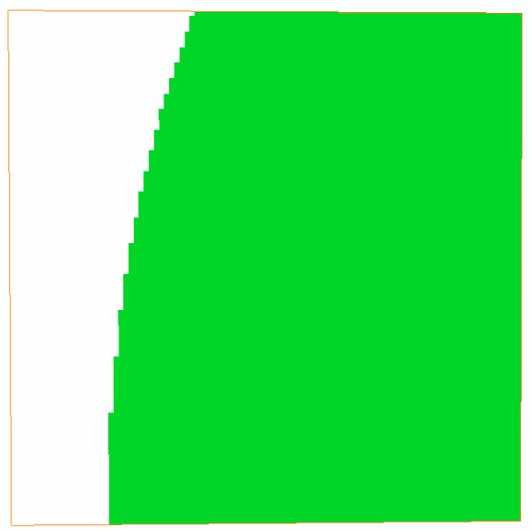

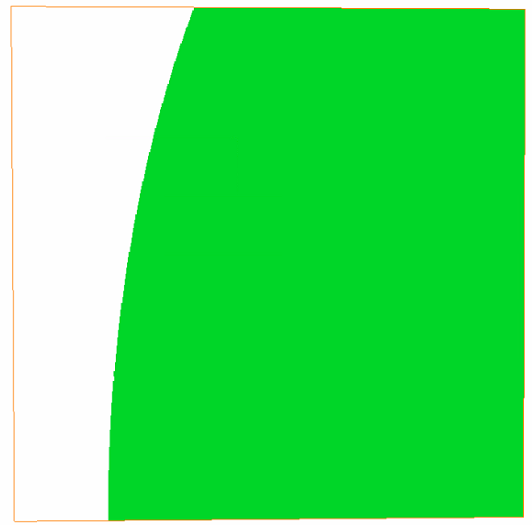


**(c) (d)**

**Figure. S2 The comparison of the 3D reconstructed result before (a) and after (b)** **cubic spline interpolation technique. (c) and (d) are one slice of (a) and (b), respectively**

**The** **optimization algorithm.**  In step 2 of the optimization algorithm, the model is optimized with the pore-size distribution obtained from experiment. Now we show some specific show the effect of the results to optimization algorithm. The pore-size distribution at different iteration during step 2 are shown in Fig. S3. Further, they are compared with the experimental data from nitrogen adsorption. It can been obtained from the figure that as the number of iterations increases, the pore-size distribution of the reconstructed result are approach to the experimental data. For more detail of the optimization of the pore-size distribution, please see the reference [5].

**Figure. S3 The pore-size distribution obtained from the reconstructed models at 100, 1000 and 3597 iterations.**

**Supplementary Reference List**

1. Pan, Z.J., Ma, Y., Connell L.D., Down, D.I.&Camilleri, M. Measuring anisotropic permeability using a cubic shale sample in a triaxial cell. *Journal of Natural Gas Science and Enginering*. **26**, 336–344(2015).
2. Wu, T.H., Li, X., Zhao, J. L.&Zhang, D.X. Multiscale pore structure and its effect on gas transport in organic-rich shale. Water Resources Research. 53,5438–5450 (2016)
3. Jiang, W.B., Lin, M., Yi, Z.X., Li, H.S.&Wu, S.T. Parameter Determination Using 3D FIB-SEM Images for Development of Effective Model of Shale Gas Flow in Nanoscale Pore Clusters. Transp Porous Med. 117,5-25(2017)
4. Darabi, H., Ettehad, A., Javadpour, F. & Sepehrnoori, K. Gas flow in ultra-tight shale strata. *J. Fluid Mech*. **7**, 641–658 (2012)
5. Ji, L.L., Lin, M., Jiang, W.B.&Cao, G.H. A hybrid method for reconstruction of three-Dimensional heterogeneous porous media from two-dimensional images. Journal of Asian Earth Sciences. Doi:https://doi.org/10.1016/j.jseaes.2018.04.026
